# Identifying novel genes in *C. elegans *using SAGE tags

**DOI:** 10.1186/1471-2199-11-96

**Published:** 2010-12-10

**Authors:** Matthew J Nesbitt, Donald G Moerman, Nansheng Chen

**Affiliations:** 1 Department of Molecular Biology and Biochemistry, Simon Fraser University, Burnaby, British Columbia, Canada; 2 Department of Zoology, University of British Columbia, Vancouver, British Columbia, Canada

## Abstract

**Background:**

Despite extensive efforts devoted to predicting protein-coding genes in genome sequences, many *bona fide *genes have not been found and many existing gene models are not accurate in all sequenced eukaryote genomes. This situation is partly explained by the fact that gene prediction programs have been developed based on our incomplete understanding of gene feature information such as splicing and promoter characteristics. Additionally, full-length cDNAs of many genes and their isoforms are hard to obtain due to their low level or rare expression. In order to obtain full-length sequences of all protein-coding genes, alternative approaches are required.

**Results:**

In this project, we have developed a method of reconstructing full-length cDNA sequences based on short expressed sequence tags which is called *s*equence *t*ag-based *a*mplification of *c*DNA *e*nds (STACE). Expressed tags are used as anchors for retrieving full-length transcripts in two rounds of PCR amplification. We have demonstrated the application of STACE in reconstructing full-length cDNA sequences using expressed tags mined in an array of serial analysis of gene expression (SAGE) of *C. elegans *cDNA libraries. We have successfully applied STACE to recover sequence information for 12 genes, for two of which we found isoforms. STACE was used to successfully recover full-length cDNA sequences for seven of these genes.

**Conclusions:**

The STACE method can be used to effectively reconstruct full-length cDNA sequences of genes that are under-represented in cDNA sequencing projects and have been missed by existing gene prediction methods, but their existence has been suggested by short sequence tags such as SAGE tags.

## Background

The nematode *Caenorhabditis elegans*, which is a well-established model organism for biomedical research [1], is the first metazoan whose genome was subject to whole-genome sequencing [2]. Its gene models were first predicted using the gene prediction program Genefinder (P. Green, unpublished). Over the dozen years since the completion of the *C. elegans *genome sequencing project [2], the *C. elegans *gene set has been curated by the *C. elegans *research community and by WormBase curators [1,3-5]. However, the *C. elegans *gene set is still far from complete for the following reasons: First, because Genefinder, like other gene prediction programs, was developed based on an incomplete understanding of gene structures, it suffers from both false positive and false negative predictions; second, many *bona fide *genes, especially those of unknown character, have been missed. In WormBase http://www.wormbase.org, the official database for the biology and genomics of *C. elegans*, less than 40% of the annotated gene models are fully confirmed. All others are either partially supported or not supported at all. Additional gene models have been revealed in transcriptome sequencing [6,7], suggesting many gene models remain to be discovered. This situation is also true for other species [8]. In the human genome, it has been estimated that the most accurate programs only correctly predict 40% of the annotated genes [9].

In this project, we explored how to reconstruct full-length gene models for genes that are not correctly represented in the current gene set, using expressed sequence tags obtained in large-scale gene expression projects. In particular, we attempted to reconstruct novel *C. elegans *gene models using SAGE (serial analysis of gene expression). The SAGE technique was originally developed for profiling gene expression [10,11]. The expression profiles created with SAGE have a wide range of applications that include therapeutic target identification in cancerous tissues [12] and others of biological and medical importance [13]. Recently, SAGE was applied to probe gene expression in *C. elegans *by the *C. elegans *Gene Expression Consortium http://elegans.bcgsc.bc.ca/home/ge_consortium.html. These SAGE libraries have been fundamental for the success of a variety of research projects [14-19]. While SAGE tags that correspond to existing gene models can be used to evaluate the abundance of gene expression, there are a large number of SAGE tags that do not correspond to existing gene models. These SAGE tags suggest the existence of additional coding exons, splice variants [20], or novel genes.

## Results

### Tag based reconstruction of full-length cDNA sequence of novel genes

Expressed sequence tags that cannot be aligned to the *C. elegans *virtual transcriptome (i.e., cDNA sequences of all annotated transcripts) suggest the existence of yet unannotated genes [13,21]. We have established a protocol, termed as "sequence *t*ag-based *a*mplification of *c*DNA *e*nds", or STACE, based on the RACE protocol [22], to identify potential novel genes. The method can be used to amplify full-length cDNA transcripts that have been reverse-transcribed from the mRNA sequence of novel genes. STACE uses three primer hybridization sites. The first site (the 5' site) is a sequence located at the extreme 5' end of the target transcript, the second site (the 3' site) is downstream of the polyadenylation sequence, and the third site (the gene-specific site) corresponds to the genomic span where the uncharacterized tag maps. The amplicons are then cloned, sequenced and mapped to the genome. As such, STACE not only confirms the existence of a novel gene, but also defines the full-length transcript sequence of the yet undefined gene.

In this project, in order to get a primer hybridization site at the extreme 5' end of the RNA transcripts, we took advantage of the trans-splice leader 1 (SL1) in *C. elegans*, and used its sequence as a primer for our 5' site. It is appropriate to design the 5' primer based on the SL1 sequence because SL1 is trans-spliced to the extreme 5' end of nearly 50% of all *C. elegans *mRNAs [23,24]. For applications in which the sample transcriptome does not undergo trans-splicing of this nature, a common oligo anchoring sequence can be ligated to the 5'end of each transcript. An oligo sequence was attached to the polyadenylation tracks of mRNA through reverse transcription with a modified oligo d(T) primer that included a 3' common sequence (5' - CCAGACACTATGCTCATACGACGCAGT_(16)_VN - 3'). This provided us with a cDNA library containing transcripts that had a usable 3' site. Finally, we chose gene-specific sites by bioinformatically identifying SAGE tags. When aligned to the *C. elegans *genome, qualified SAGE tags do not overlap with existing gene models. For each qualified SAGE tag, a primer was designed and used in conjunction with a primer complementary to the SL1 sequence to amplify the upstream amplicon. A second primer was designed and used in conjunction with the primer complementary to the 3' common sequence (above) to amplify the downstream amplicon. The potential template was amplified, and the amplicon sequences were mapped to the *C. elegans *genome using BLAT [25], which is available at WormBase http://www.wormbase.org.

### Computational selection of SAGE tags that suggest novel genes

SAGE tags used in this study were selected from 33 SAGE libraries, which were sequenced from different tissues and developmental stages of *C. elegans *http://tock.bcgsc.bc.ca/cgi-bin/sage160. There are altogether 220,770 unique SAGE tags in these libraries. Only SAGE tags that did not overlap with annotated protein-coding genes in the WS160 version of the *C. elegans *genome map were selected for this project.

We obtained four different sets of SAGE tags for testing, one preliminary set and three test sets (Set 1-3) (Table 1). The preliminary set, which was arbitrarily chosen, was used to test the STACE protocol. Set 1 used a longSAGE meta-library as a starting tag set (16,587 SAGE tags). Set 2 was created from the WS160 version of the mixed stage library (14,701), and Set 3 used SAGE libraries derived from Solexa sequencing of the SWN21 and SWN22 embryonic samples (359,457 SAGE tags). Solexa SAGE produced more initial SAGE tags than the previous SAGE libraries because it has much deeper coverage. Note that the Illumina Solexa Genome Analyzer produced a SAGE library that is about 20 times more sensitive than a normal SAGE library [26].

**Table 1:** SAGE tag numbers for each set through the identification of high value candidate SAGE tags.

	*Total tags*	*Mappable tags*	*Non-transcriptome tags*	*Tags with frequency count >3*	*Tags absent from gene boundaries and introns*	*Tags with appropriate GC content*	*Tags that can serve as primers*	*SAGE tag primers tested*
*Set 1*	16,587	13,743	3,052	616	418	128	39	30

*Set 2*	14,701	10,534	4,755	365	41*	19	12	12

*Set 3*	359,457	32,416	13,542	8,211	469	124	106	96

**Figure 1:**
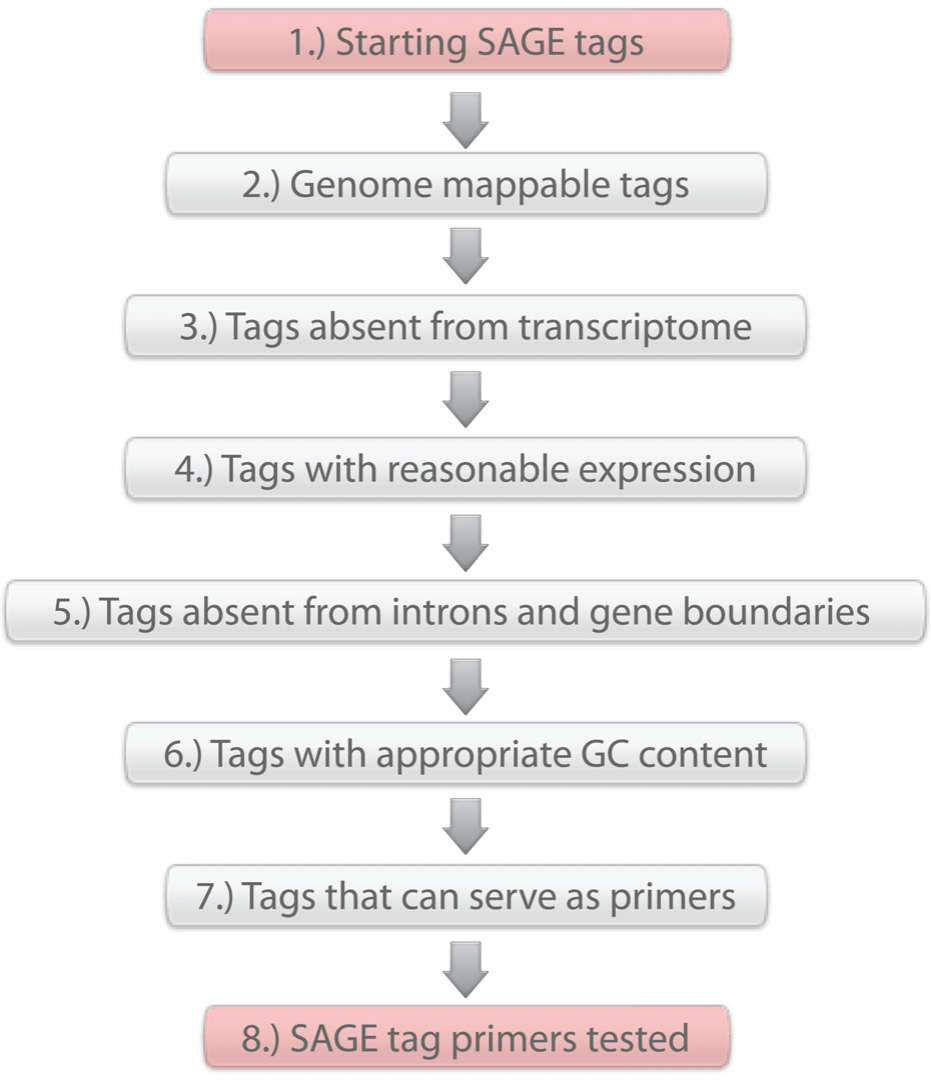
**SAGE tag primer development**. SAGE tag pools are put through various filtrations to produce a source of SAGE tag primers that are used in STACE experiments. 1) SAGE tag libraries are downloaded from the MultiSAGE website. 2) All SAGE tags that do not fully map to the genome as one single uninterrupted alignment are discarded. 3) SAGE tags that map to annotated transcribed sequences are discarded. 4) SAGE tags that have a low level of expression and are possible errors in sequencing are discarded. 5) SAGE tags that are found to overlap with intronic sequences or map near a 5' or 3' boundary are eliminated. 6) Only tags with GC content between 35% and 45% are retained. 7) SAGE tags are edited into primer form. All SAGE tags that are likely to produce secondary structure (i.e. hairpins, homo-dimers, hetero-dimers) are discarded. 8) A final list of SAGE tag primers is procured.

SAGE libraries were filtered to select SAGE tags for finding novel genes (Figure 1). The criteria used included the following: (1) Only SAGE tags that can be aligned to the *C. elegans *genome were selected; (2) The SAGE tags must not overlap with any annotated coding exon; (3) To avoid tags containing sequencing errors, SAGE tags must have a frequency of at least three for every 100,000 reads; (4) To increase the chances of finding novel genes (rather than novel missing exons), the SAGE tags must not overlap with an intron and have to be at least 500 bp away from an annotated 5' or 3' gene boundary; (5) SAGE tags must have a GC content between 35% and 45%, which is critical for primer design.

Primers based on SAGE tags were designed to ensure a reduced possibility of formation of secondary structures which would inhibit proper annealing of the primers [27]. For many cases, we trimmed sequences from either end of the SAGE tags to ensure primer quality. SAGE tag sequences that could not be used to guide proper primer design were not used. Primer design was done using the Primer3 program [28].

### cDNA libraries

Two different cDNA libraries were created; one from a mixed stage population of *C. elegans *and another one from embryonic animals. In order to maximize the number of successful experiments, candidate SAGE tags were only screened against the developmental library that corresponded with the time in development that the tags were originally observed.

### New transcripts and novel cDNAs

**Figure 2:**
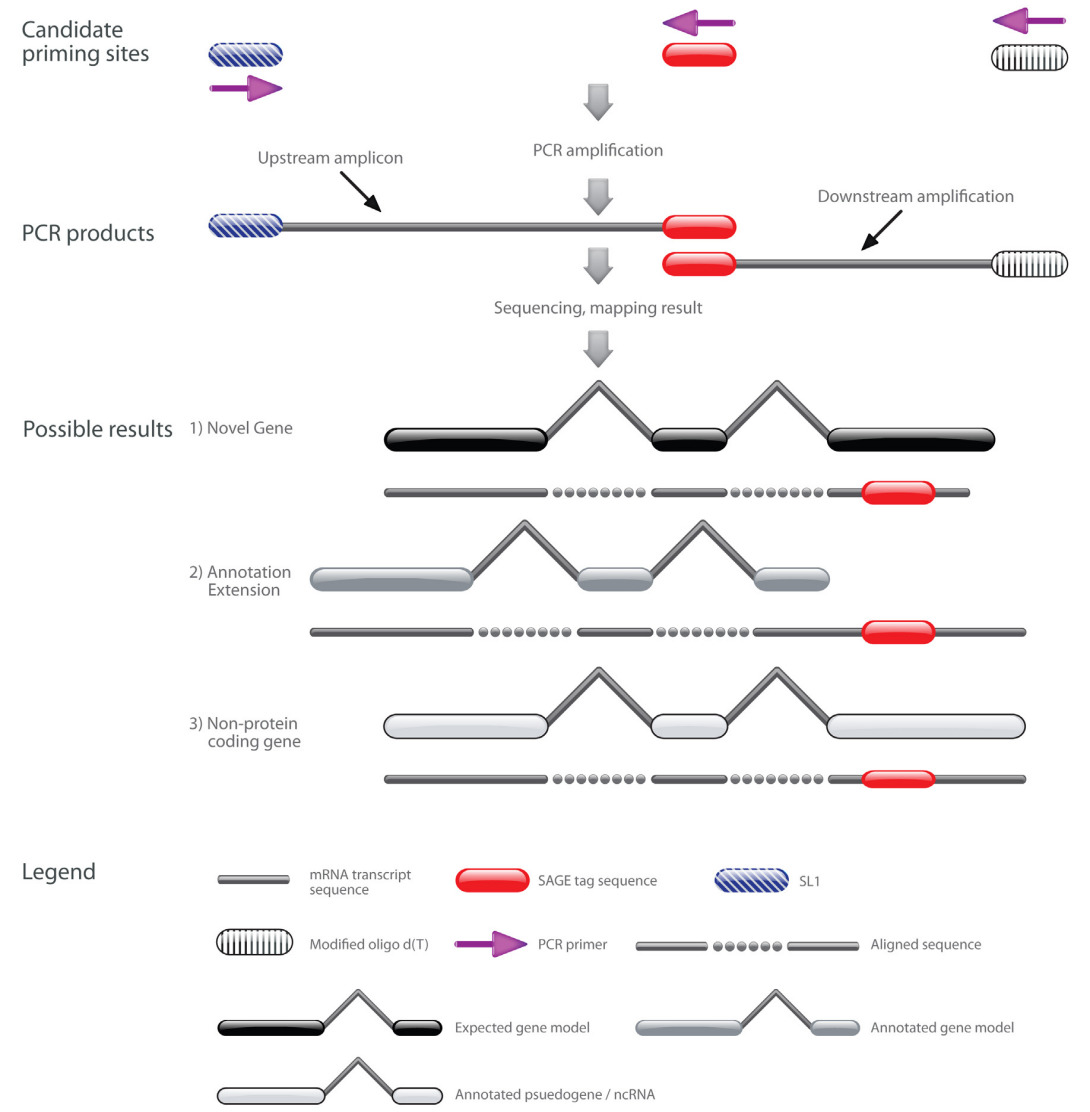
**STACE experimental procedure**. Template cDNA is used in two separate PCR reactions that utilize primers based on the sequence of a SAGE tag, the SL1 sequence and modified oligo d(T) primer. The PCRs produce an amplicon representative of the sequence upstream of the SAGE tag location (PCR product 1), and another that comes from the downstream sequence (PCR product 2). These products are sequenced, and mapped to the *C. elegans *genome. Those sequences whose alignments overlap with the SAGE tag used in primer design are considered true positive STACE results.

STACE-identified candidates consist of three categories based on the alignments of these candidates to the *C. elegans *genome (release WS160): (1) novel gene (six candidates), (2) annotation extension (four candidates), and (3) non-protein-coding gene overlap (two candidates) (Figure 2; Table 2). Novel genes are proof of entirely new genes discovered using the STACE method. The exons of these six new genes are all bordered by the canonical GT-AG splice signals, as is the case with most exons [29,30]. Our identification of six novel genes was based on using the WS160 annotated genome. In the interim four have been annotated in WS200, while the other two are still completely novel. One of these two new gene models was characterized as a full-length gene model with the STACE method, while the other's existence was implied by an upstream amplicon sequence. Four tested SAGE tag primers produced results that suggest an extension to the annotated length of the gene models. These annotated extensions align perfectly with annotated exons, and imply either additional exons are transcribed within the gene, or that the terminal exons are longer than shown by WormBase.

**Table 2:** Result classifications for all sets of tested SAGE tag primers.

	Novel Genes	Annotation Extensions	Non-Protein Gene Overlap	Number of Candidate cDNAs/Number of SAGE tag Primers Tested
Preliminary Set	1	0	2	3/6 (50%)

Set 1	3	1	0	4/30 (13%)

Set 2	0	2	0	2/12 (17%)

Set 3	2	1	0	3/96 (3%)

Total	6	4	2	12/144 (8%)

We found a successful STACE result overlapped with a pseudogene. While this transcript may not be translated, using STACE we have clearly shown that it is processed with introns removed and a polyadenylation track added to the 3'end. We have also found that a STACE result overlapped with an annotated ncRNA gene. The transcript was also processed with a previously unknown intron excised and a polyadenylation track added.

Altogether, we have reconstructed seven full-length, true positive cDNA sequences, corresponding to seven separate gene models (Table 3). All seven cDNAs contain SL1 signals at the 5' ends and polyadenylation at the 3'ends. The remaining seven true positive cDNAs recovered represent the 5'ends of separate gene models, and these too contain full-length 5' SL1 signals. Thus, in this study, we have identified 14 SL1-trans-spliced cDNA sequences. All 14 cDNA sequences have been submitted to GenBank (Table 3).

**Table 3:** Identified cDNA sequences from Set 3 STACE experiments.

*Result*	*SAGE tag primer*	*SAGE tag location*	*Sequence 5' mapping boundary*	*Sequence 3' mapping boundary*	*GenBank accession number*	*Status (as of WS200)*
Full-length cDNA P.1	GTTAGGATCGTAGAGGACATG	II:8786920	II:8786297	II: 8787044	HQ451870	Overlaps F07H5.4 (pseudogene): evidence for extension to annotated exon

Partial cDNA P.2	AGAGGATTAATTCCCCCCATG	II:9375813	II:9376228	II:9375792	HQ451877	Overlaps with C06C3.10

Full-length cDNA P.3	GGGGGAAAATCGAAAGACATG	II:10201160	II: 10202155	II:10201084	HQ451871	Overlaps with tts-2 (ncRNA): evidence for new intron

Partial cDNA 1.1	GAAACGAAGAAGAAAAGCATG	V:19434698	V:19434352	V:19434718	HQ451878	Evidence of a novel gene

Full-length cDNA 1.2	TTCGACGGCAGATTGTTCATG	V:19432707	V:19433037	V:19432406	HQ451872	Overlaps with C25F9.11: evidence for new 5' UTR

Full-length 1.3	TAGCTCAGTCAAAACAACATG	V:5812559	V:5813070	V:5812296	HQ451873	Overlaps with ZC250.4: evidence for extension to 3' UTR

Partial cDNA 1.4a	AAAGTTGAGCTTCTGCTCATG	X:2346863	X:2335678	X:2346883	HQ451879	Overlaps with T01B6.1: evidence for new coding sequence

Partial cDNA 1.4b	AAAGTTGAGCTTCTGCTCATG	X:2346863	X:2345479	X:2346883	HQ451880	Overlaps with T01B6.1: evidence for new transcriptional start site

Partial cDNA 2.1a	TGGTTGTTAGTAGTGTACATG	II:15229391	II:15207408	II:15229412	HQ451881	Overlaps with Y46E12BL.4: evidence for new 3' UTR exon

Partial cDNA 2.1b	TGGTTGTTAGTAGTGTACATG	II:15229391	II:15216289	II:15229412	HQ451882	Overlaps with Y46E12BL.4: evidence for new initial coding exon

Full-length cDNA 2.2	CCATCTAAAGGGCTCTACA	IV:4415359	IV:44085996	IV:4415616	HQ451874	Overlaps with Y24D9A.1: evidence for extension to 3' UTR

Full-length cDNA 3.1	CTCATTGAAGGTGAAGCAT	X:14690913	X:14692920	X:14690763	HQ451875	Overlaps with sox-3: evidence for new 3' UTR

Partial cDNA 3.2	TGAAATGTCACAGTACACAT	III:7604002	III: 7601399	III:7604022	HQ451883	Evidence of a novel gene

Full-length cDNA 3.3	GAGAGAATTGTTGTGACCAT	X:4681136	X:4682689	X:4680952	HQ451876	Evidence of a novel gene

All data is current as of WS200.

## Discussion

**Figure 3:**
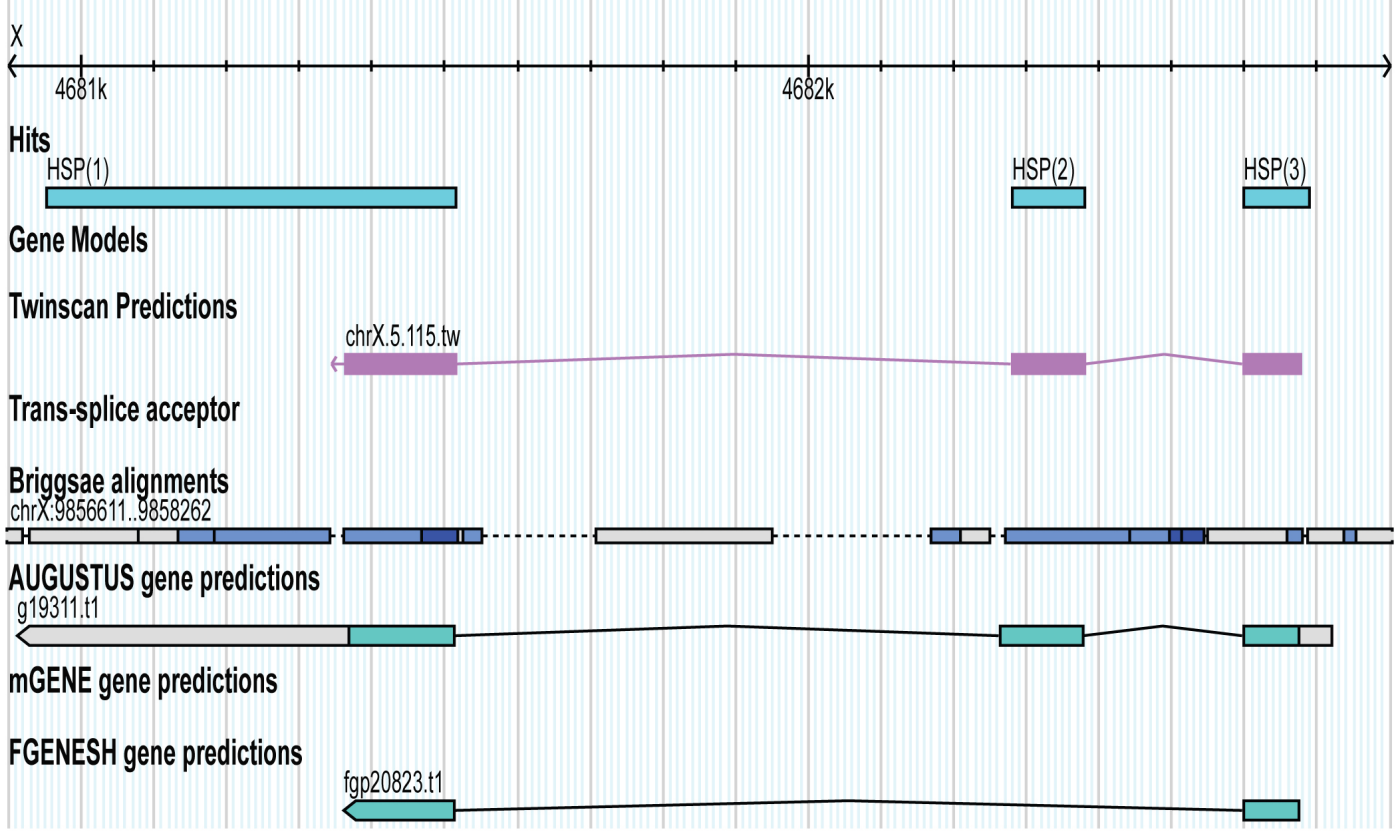
**Candidate cDNA 3.3 alignment**. Full length gene model reconstructed for candidate cDNA 3.3. This gene model suggests a completely novel gene that is missing from WormBase as of WS200.

**Figure 4:**
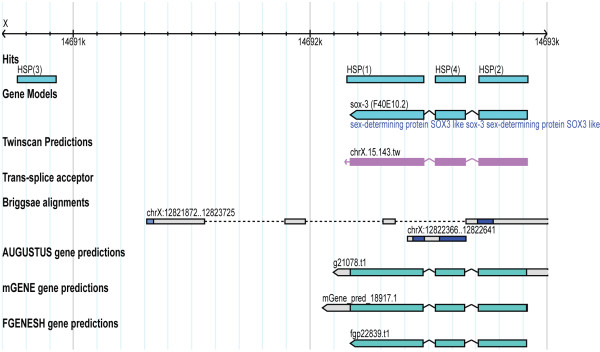
**Candidate DNA 3.1 alignment**. Full length gene model reconstructed for candidate cDNA 3.1. This gene model suggests a 3' UTR extension to the current sox-3 gene model.

**Figure 5:**
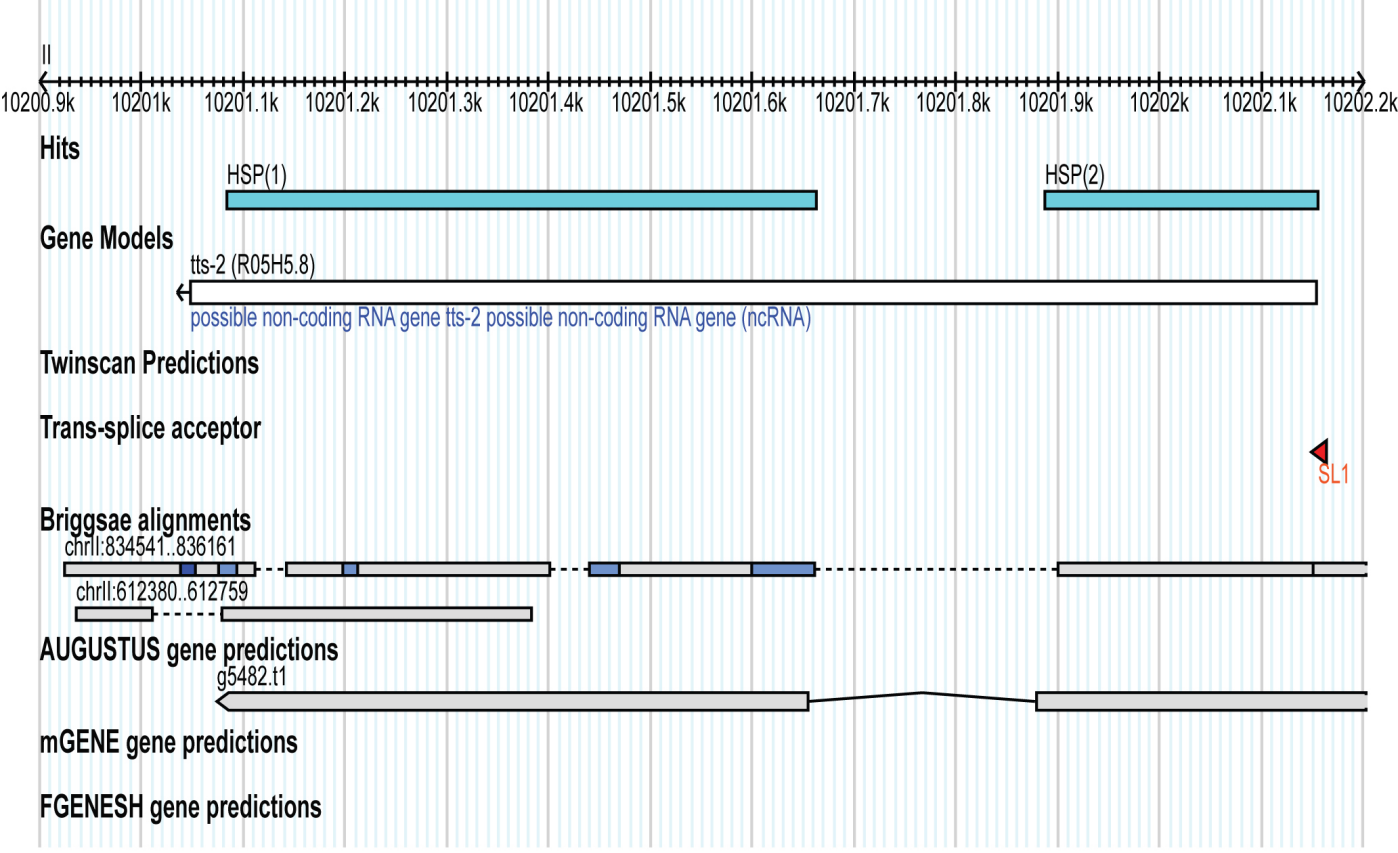
**Candidate cDNA P.3 alignment**. Full length gene model reconstructed for candidate cDNA P.3. This gene model suggests revision of the structure to the tts-2 gene model, and also that this transcript is polyadenylated, a feature that is commonly associated with protein-coding genes.

In this project, we have developed an experimental method termed STACE for reconstructing full-length cDNAs of novel genes. The applicability of STACE has been demonstrated by defining novel genes in the well-curated *C. elegans *genome, using SAGE tags from gene expression studies. We reconstructed seven novel full-length cDNAs and seven partial cDNA sequences that can be merged to existing gene models. Novel genes, annotation extensions, and non-protein-coding gene overlaps are represented by the identified cDNA sequences 3.3 (Figure 3), 3.1 (Figure 4), and P.3 (Figure 5), respectively.

We compared novel cDNAs with *C. elegans *gene models predicted using AUGUSTUS [31], mGENE [32], TWINSCAN [33] and FGENESH++ [34], which are available at WormBase. All cDNAs, which were detected using STACE, when aligned to the *C. elegans *genome overlap to a certain extent with predicted gene models. The novel full-length cDNA 3.3 aligned well with a prediction from TWINSCAN and with a prediction made by FGENESH++. The annotation extension result (full-length cDNA 3.1) was found to overlap with gene predictions from each of the utilized programs. However, a new 3' UTR exon was shown to be part of this gene model, and this exon did not overlap with the predictions made by any of the described programs. Additionally, the P.3 result overlapped with an existing ncRNA gene model. However, the novel intron suggested by this STACE result was not included in the WormBase gene model, although it overlaps with AUGUSTUS prediction.

## Conclusions

We have found that the STACE method can be used to recover accurate full-length gene models. This method is useful for reconstructing gene models for genes that have been missed in cDNA sequencing projects and were missed or mispredicted by gene finders. With the wide application of next-generation sequencing methods in the deep sequencing of transcriptomes, more expressed sequence tags, which indicate the presence of novel genes will be uncovered. We expect that these tags will serve as input to the STACE protocol for further novel gene discovery and determination.

## Methods

### cDNA library production

Two samples of *C. elegans *were produced that represented both a mixed stage population and an embryonic sample. Tissue samples were put through an RNA extraction using TRIzol (Invitrogen, SKU# 10296-028). The cDNA libraries used in this project were created with the Superscript III reverse transcriptase kit (Invitrogen, SKU# 18080-085), and the primer used to initiate reverse transcription was a modified oligo d(T) primer (5' - CCAGACACTATGCTCATACGACGCAGT_(16) _VN - 3') (Invitrogen). The protocol accompanying the kit was followed, and the samples were treated with Ribonuclease H (Invitrogen, SKU# 18021-014).

### Amplification of tag ends

The reverse complement of each SAGE tag sequence was used to design the SAGE tag primers. These primers were used in conjunction with a primer based on the SL1 sequence (5' - GGTTTAATTACCCAAGTTTGAG - 3') in a PCR. The PCR was initiated with a 94°C melt step for 2 minutes, followed by 32 cycles of a 94°C melt step for 15 seconds, a 60°C annealing step for 45 seconds, and a 72°C extension step for 1 minute. This was followed by a final extension at 72°C for 5 minutes. A Taq polymerase provided by Dr. Harald Hutter was used in all of the PCRs. Amplicons produced by the PCRs were visualized with a 1% gel electrophoresis, and extracted with a QIAquick Gel Extraction kit (Qiagen, ID 28704). These amplicons were then cloned with the InsTAclone kit (Fermentas, #K1214). Cloned amplicons were submitted for sequencing (Macrogen, Seoul, Korea), and returned sequences were mapped back to the *C. elegans *genome with the BLAT tool [25] on the WormBase website http://www.wormbase.org/. We opted to use BLAT instead of other alignment tools because this program can take spliced mRNA sequences (i.e. STACE cloned sequences) and align them to the genome in a way that reflects intron - exon boundaries [25,35]. Those amplicons whose sequence alignment indicated a true positive result were then further studied. The returned sequence was used to design an internal primer that would be compatible with the universal primer (5' - CACTATGCTCATACGACGCAGT - 3'). These primers were then used in a PCR with the same parameters described above to produce the downstream amplicons needed for full-length characterization. Internal primers were designed using the Primer3 program [28].

## Authors' contributions

NC and DGM conceived of the study. MJN conducted the experiments. MJN and NC wrote the manuscript with input from DGM. All authors have read and approved the final manuscript.
